# Spare *PRELI* Gene Loci: Failsafe Chromosome Insurance?

**DOI:** 10.1371/journal.pone.0037949

**Published:** 2012-05-30

**Authors:** Wenbin Ma, Morgan R. McKeller, Roberto Rangel, Blanca Ortiz-Quintero, Michael R. Blackburn, Hector Martinez-Valdez

**Affiliations:** 1 Department of Immunology, The University of Texas M.D. Anderson Cancer Center, Houston, Texas, United States of America; 2 Department of Biochemistry and Molecular Biology, The University of Texas-Houston Medical School, Houston, Texas, United States of America; National Institutes of Health, United States of America

## Abstract

**Background:**

*LEA* (late embryogenesis abundant) proteins encode conserved N-terminal mitochondrial signal domains and C-terminal (A/TAEKAK) motif repeats, long-presumed to confer cell resistance to stress and death cues. This prompted the hypothesis that *LEA* proteins are central to mitochondria mechanisms that connect bioenergetics with cell responses to stress and death signaling. In support of this hypothesis, recent studies have demonstrated that mammalian *LEA* protein *PRELI* can act as a biochemical hub, which upholds mitochondria energy metabolism, while concomitantly promoting B cell resistance to stress and induced death. Hence, it is important to define *in vivo* the physiological relevance of *PRELI* expression.

**Methods and Findings:**

Given the ubiquitous PRELI expression during mouse development, embryo lethality could be anticipated. Thus, conditional gene targeting was engineered by insertion of flanking loxP (flox)/Cre recognition sites on *PRELI* chromosome 13 (Chr 13) locus to abort its expression in a tissue-specific manner. After obtaining mouse lines with homozygous PRELI floxed alleles (*PRELI* ^f/f^), the animals were crossed with CD19-driven Cre-recombinase transgenic mice to investigate whether *PRELI* inactivation could affect B-lymphocyte physiology and survival. Mice with homozygous B cell-specific *PRELI* deletion (CD19-Cre/Chr13 *PRELI* ^−/−^) bred normally and did not show any signs of morbidity. Histopathology and flow cytometry analyses revealed that cell lineage identity, morphology, and viability were indistinguishable between wild type CD19-Cre/Chr13 *PRELI* ^+/+^ and CD19-Cre/Chr13 *PRELI* ^−/−^ deficient mice. Furthermore, B cell *PRELI* gene expression seemed unaffected by Chr13 *PRELI* gene targeting. However, identification of additional *PRELI* loci in mouse Chr1 and Chr5 provided an explanation for the paradox between *LEA*-dependent cytoprotection and the seemingly futile consequences of Chr 13 *PRELI* gene inactivation. Importantly, *PRELI* expression from spare gene loci appeared ample to surmount Chr 13 *PRELI* gene deficiency.

**Conclusions:**

These findings suggest that *PRELI* is a vital *LEA* B cell protein with failsafe genetics.

## Introduction

In attempting to understand how mitochondria bioenergetics is biochemically connected with stress and death signaling, recent studies found that *PRELI* (protein of relevant evolutionary and lymphoid interest) [1], [2] is a key mammalian *LEA* ortholog inherently involved in mechanisms that regulate mitochondria biogenesis, energy metabolism, and cell survival [3], [4], [5]. Importantly, *PRELI* was shown to interact with dynamin-like GTPase *OPA1* [6] to keep cristae junctions intact, control molecular current [5], [7], and support critical biochemical mitochondria functions. For instance, mitochondria convert organic compounds into CO_2_ and H_2_O to produce energy in the form of ATP, via respiratory chain (RC) oxidative phosphorylation reactions [5], [7], [8], [9]. These reactions were found to be enhanced by *PRELI* expression, thus enabling RC progression from complex I to V and ensuring the maintenance of the mitochondrial membrane potential (Δψ_m_) [5], [10], [11]. Notably, the mitochondrial Δψ_m_ supports energy metabolism, regulates reactive oxygen species (ROS) production and controls the traffic of pro- and anti-apoptotic molecules [5], [12], [13]. As such, the mitochondrial Δψ_m_ becomes a conductance core, where crosstalk between bioenergetics and apoptosis signaling directs cell responses and fate [14].

In keeping with this notion, *LEA* protein-dependent maintenance of the mitochondrial Δψ_m_ is essential to prevent ROS surges, promote the assembly of survival protein networks and restrain the release of hallmark contributors of programmed and induced cell death [5], [12], [14], [15], [16]. Remarkably, the intrinsic mitochondria-dependent cytoprotection functions that are relevant to *LEA*-containing proteins like *PRELI*, have been documented by studies on plants and vertebrate and invertebrate animals, in which evolutionary parallels of cell responses to stress and death-inducing stimuli are well established [3], [5], [17], [18], [19], [20], [21]. Furthermore, yeast (*Ups1p*) and mammalian *PRELI* locate within mitochondria inter-membrane space (IMS), interact with respective yeast *Mgm1p* or mammalian *OPA1* at cristae junctions, and play analogous roles in mitochondria biogenesis and cell survival [5], [17], [22]. Moreover, human and mouse *PRELI* proteins are 96.3% identical and exhibit robust and ubiquitous expression during embryo development [1], [2], which supports the notion of evolutionarily conserved mechanisms for eukaryote cell development and survival [3], [4], [5], [17], [23].

Therefore, to directly assess the physiological significance of mammalian *LEA*-containing proteins *in vivo*, we sought to conditionally target the mouse Chr13 *PRELI* gene, the only known locus at the time these studies were undertaken. The findings reported herein and the recent disclosure of additional *PRELI* gene copies in Chr1 and Chr5 provide evidence that failsafe mechanisms are genetically imprinted to ensure the expression of genes that are necessary for cell survival.

## Results

### Protein structure features defining PRELI physiology

**Figure 1:**
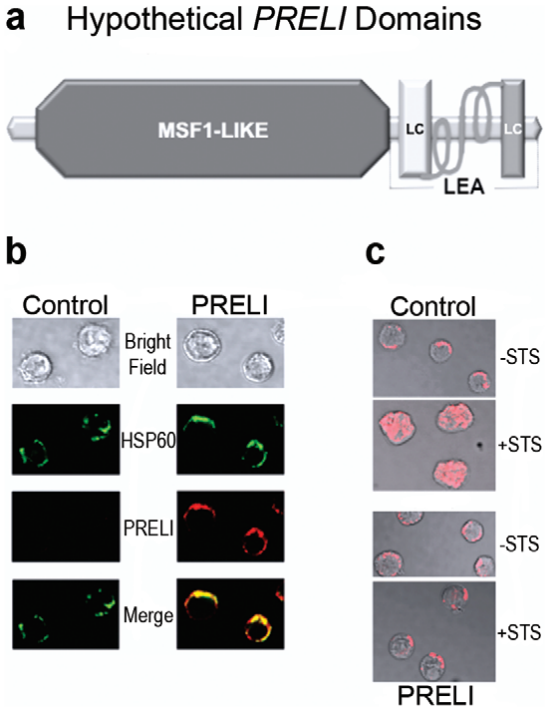
*PRELI* motifs and their relevance in subcellular location and function. (**a**) Hypothetical *PRELI* structure based on queries of deduced amino acid sequence against protein databases (http://wwwuniprot.org/uniprot/Q8R107; http://pfam.sanger.ac.uk/family/PF04707). *PRELI* MSF1-like region is depicted as a dark grey octagonal form, while its *LEA* motif is shown as theoretical α helices, flanked by low complexity (LC) domains (lighter grey rectangles). (**b**) Confocal microscopy results on control (Vector) and *PRELI* PBlin-1 transfectants [5] confirm MSF1-like deduced prediction that N-terminal signal peptide **mvkyflgqsvlrsswdqvfaafwqrypnpyskhvl** can direct *PRELI* expression (red fluorescence) into the mitochondria, as shown by its co-localization (merge) with mitochondrial HSP60 (green fluorescence) [5]. (**c**) Confocal microscopy results show that in contrast to control cells (Control), *PRELI* Blin-1 transfectants (*PRELI*) [5] prevent STS-induced (+STS) release of *AIF* from the mitochondria and uphold cell morphology and survival. These results are consistent with the conserved cytoprotection function of *LEA*-containing proteins. *AIF* is shown in red fluorescence within greyscale cell background.

As mammalian *LEA* proteins, human and mouse *PRELI* display conserved MSF1-like domains and C-terminal coiled-coil (CC) *LEA* motif (*A/TAEKAK*) repeats [2], [3], [5], [23]. The hypothetical MSF1-like domain (Fig. 1a) spans a 170 amino acid module, which is known to assume globular α/β conformations and harbor pleckstrin homology (PH) regions, phosphothyrosine binding (PTB) sites, epsin N-terminal homology (ENTH) motifs and 4.1 Ezrin-Radixin-Moesin (FERM) domains [24], [25]. These structures are characteristic of signal transduction proteins involved in the regulation of mitochondrial molecular traffic [24], [25]. Relevant to PRELI mechanisms of action, the MSF1-like sequence comprises N-terminal mitochondria localization signals [26]. Thus, to further attest the relevance of the MSF1-like structure in defining *PRELI* expression in the mitochondria, control (Vector) and *PRELI* Blin-1 transfectants were comparatively analyzed by immunofluorescence staining and confocal microscopy. Consistent with *PRELI* N-terminal structure, Figure 1b reveals a selective expression of the mammalian *LEA* protein, in the mitochondria by *PRELI* Blin-1 transfectants. Markedly, the parental Pre-B acute lymphocytic leukemia (PreB ALL) Blin-1 cell line [27] did not express detectable *PRELI* levels, which underscored its sensitivity to caspase-dependent and independent apoptosis [5].

Equally important to *PRELI* structure-dependent features, the tandem *LEA* (*A/TAEKAK*) repeats are configured as two α helices in opposite orientation, which are flanked by low complexity (LC) regions (Fig. 1a) and characterize proteins that confer stress tolerance and resistance to death [3], [5], [18], [21], [28]. In agreement with this notion and in further support of earlier findings [5], figure 1c shows that *PRELI* expression is essential to confine apoptosis inducing factor (*AIF*) [29] in the mitochondria and thus prevent caspase-independent death. Notably, *AIF* release from the mitochondria results in direct DNA fragmentation and virtually immediate cell destruction [5]. Thus, the potent *LEA* protein-dependent cytoprotection was emphasized by the evidence that in contrast to control Blin-1 transfectants (Fig. 1c top), the imminent and devastating death resulting from *AIF* redistribution was largely preventable in *PRELI* expressing cells (Fig. 1c, bottom).

### *PRELI* expression during mouse embryo development

**Figure 2:**
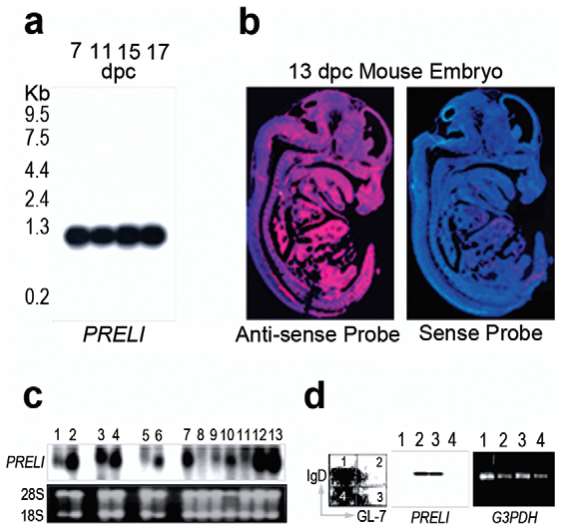
*PRELI* mRNA expression during mouse embryo development and in adult life. (**a**) Northern blot results show the prominent *PRELI* mRNA expression at 7–17 days post coitum (dpc) stages of mouse embryo development. (**b**) *In situ* hybridization results of whole-mount embryo sections show robust and ubiquitous *PRELI* mRNA expression at 13-dpc developmental stage. Left: hybridization with digoxigenin-labeled anti-sense *PRELI* complementary RNA (cRNA) probe shows prominent red fluorescence overlapping with Hoechst blue fluorescence counterstain. Right: hybridization with sense cRNA probe, which largely exhibits the blue fluorescence of the counterstain, serves as control for *PRELI* mRNA detection specificity. (**c**) Northern blot results show *PRELI* expression by distinct adult mouse tissues: bone marrow (1), thymus (2), spleen (3), lymph node (4), testis (5), ovary (6), brain (7), skeletal muscle (8), heart (9), stomach (10), liver (11), lung (12) and kidney (13). Ethidium bromide-stained images of 28S and 18S ribosomal RNA bands, retained on nitrocellulose blots after capillary transfer, are shown as control for RNA sample loading (**d**) Left: Cell sorting scheme used for RNA isolation from naïve IgD^+^GL-7^−^ (1), germinal center (GC) precursors IgD^+^GL7^+^ (2), GC IgD^−^GL-7^+^ (3), and memory IgD^−^GL-7^−^ (4) B cell subsets. Right: RT-PCR results reveal *PRELI* mRNA expression by the distinct B cell subsets. Glyceraldehyde 3-phosphate dehydrogenase (*GAPDH*) mRNA amplification is presented as control of B cell subset RNA content.

*LEA* gene expression in all eukaryote studied is selectively prominent during embryo development and when organisms are exposed to stress and/or life-threatening conditions [3], [5], [18], [20], [21], [23], [30]. This suggested that *in vivo*, *PRELI* expression could be crucial for mammalian embryo development and when cell survival is intrinsically connected with mitochondria mechanisms that control stress tolerance and resistance to death [5]. In support of this hypothesis, northern blot and fluorescent *in situ* hybridization (FISH) analyses showed that *PRELI* mRNA expression was robust and ubiquitous throughout all mouse embryo stages (Fig. 2a and b). Although northern blot results depicted strong mRNA levels at 7, 11, 15, and 17 days post coitum (dpc), which vitually overlapped with the FISH data, the former aimed to verify *PRELI* mRNA molecular size while the latter intended to mainly illustrate the overall tissue distribution of the transcript. Moreover, the 13-dpc FISH image revealed a developmental stage not represented in the northern blot results and thus complemented *PRELI* expression analyses. Of note, the prominent 1.1 kb mRNA size detected by northern blotting, is the only mouse transcript that has been routinely confirmed by annotated sequencing data (National Center for Biotechnology Information and The European Molecular Biology Organization databases). Therefore, the weak but noticeable 7.5 kb mRNA bands detected at 11 and 15 dpc are likely the result of unprocessed transcripts. These collective results parallel *LEA* gene expression traits found in most eukaryotes.

### *PRELI* mRNA tissue distribution in adult mice

Earlier studies have shown that *LEA* proteins in general and *PRELI* in particular connect mitochondria bioenergetics with mechanisms that are essential for cell survival [3], [5], [31]. In keeping with this notion, *PRELI* mRNA tissue distribution in adult mice was found to be predominant in the thymus, spleen, lymph nodes, brain, lungs, and kidneys (Fig. 2c). These are tissues in which cells are under rigorous selection pressure [32], [33], [34] and mitochondria-dependent functions drive cell responses to stress and death stimuli [35], [36]. For instance: (**a**) Lymphocyte maturation programs in the thymus, spleen and lymph nodes entail stringent selection mechanisms that ensure the survival of immunocompetent cells and elimination of aberranly developed cells [32], [33]; (**b**) Similarly, high energy-driven mechanisms are central for brain cell survival [35]; (**c**) Lung cell survival is constantly challenged by oxidative toxicity and pathogen-driven inflammatory strain [34]; (**d**) In kidney cells, strenuous Na^H+^/Cl^e−^ exchanges are characteristic functions that sustain tissue hydration, osmolarity and survival, which parallel bioenergetic H^+^/e^−^ gradients that protect plant cells from pH collapse, desiccation, and death [19], [28], [30]; (**e**) Lastly, like immune cells, germ cells in the ovary undergo maturation selection cycles, which result in the ovulation of the fit and the demise of cells failing maturation demands [36].

It is noteworthy that while mitochondria bioenergetics is inherently associated to cellular activity in the testis, heart, skeletal muscle and liver, *PRELI* mRNA levels were virtually undetectable in these organs under homeostatic contitions (Fig. 2c). However, it is conceivable that *PRELI* expression is most prominently required when energy demands are intrinsically linked to cell responses to stress death signaling [5]. This suggests that *PRELI* expression could be under strict genetic and/or epigenetic regulatory constraints and hence, transcriptional activation could also depend on stress-mediated stimulation, including hypoxia, hypertension or inflammatory reactions.

In added support of the physiological connection of stress and death signaling with *PRELI* expression and mechanisms of cell survival, spleen and lymph node germinal center (GC) B cells are programmed to die unless they can recognize pathogen determinants (also known as antigens) with high affinity [32]. Thus, GC B cell affinty maturation is a stringent selection process and timely *PRELI* expression could be required to ensure the survival of high-affinty/antigen-binding GC B cells. Consistent with this hypothesis, figure 2d shows that *PRELI* expression is selectively expressed by GC precursors and GC B cells.

Together, these data suggest that similar to most *LEA* genes, *PRELI* expression is necessary for embryo development and important for stress tolerance and resistance to programmed and induced death in adult mice. Therefore, gene inactivation experiments were sought to appraise *in vivo PRELI* physiology.

### Conditional *PRELI* gene targeting in mice

**Figure 3:**
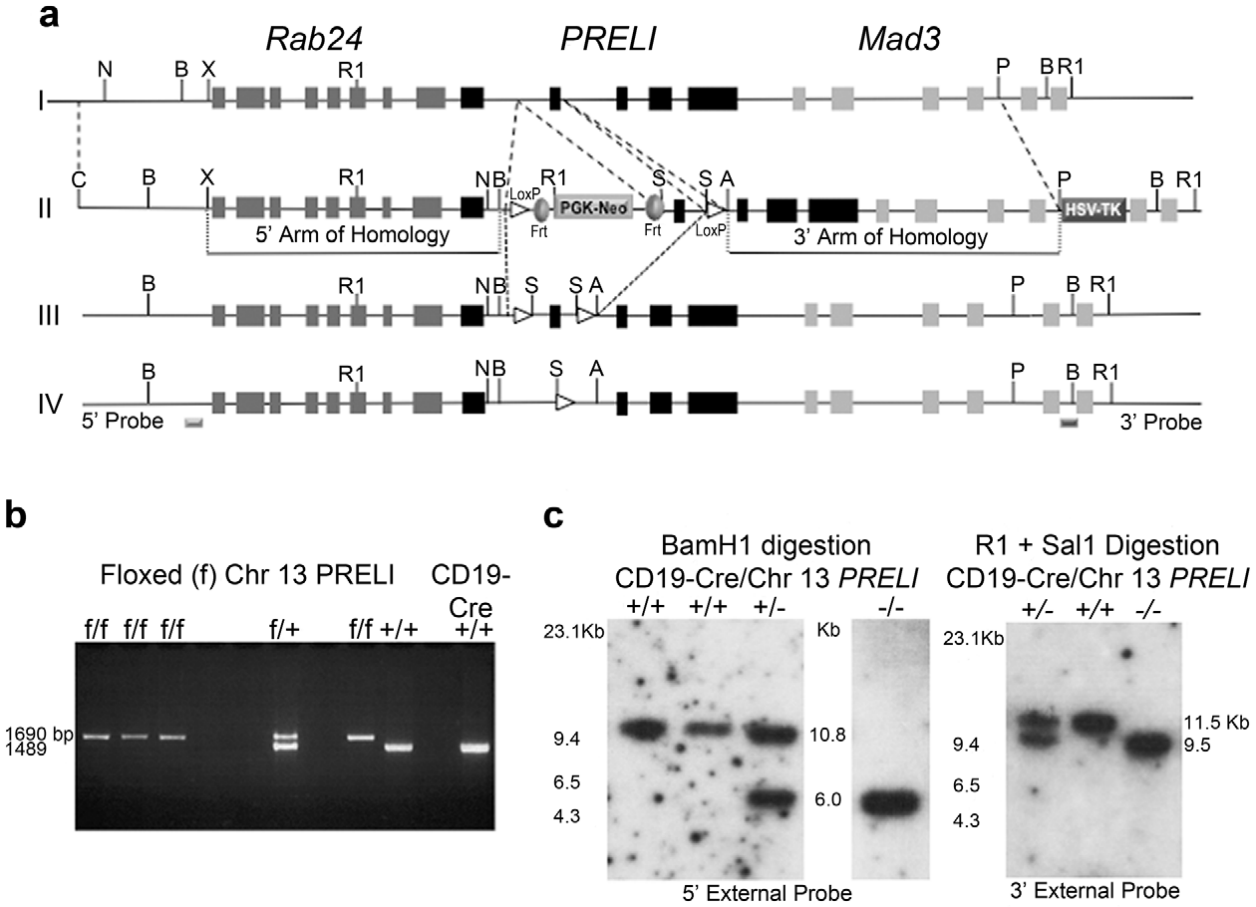
Conditional Chr 13 *PRELI* gene Targeting. (**a**) Overall Schema **I**: WT allele depicts *PRELI* gene flanked by *Rab 24* (left) and *Mad3* (right) genes; **II**: Targeting vector construct displays relevant restrictions sites (Asc1 [A], BamH1 [B], Cla1 [C], EcoR1 [R1], Not1 [N], Pme1 [P], Sal1 [S], and Xba1 [X]). Also indicated are 5′ and 3′ arms of homology, LoxP sites, Flip recombination (Frt) sequences, and PGK-Neo and HSV-Tk selection cassettes; **III**: Floxed *PRELI* allele, after Frt-mediated removal of selection cassettes; **IV**: *PRELI* deficient allele after CD19-driven Cre recombinase (Cre) cleavage of *PRELI* exon II. Location of 5′ and 3′ external probes used in Southern Blot hybridization experiments is indicated. (**b**) PCR genotyping results of mouse litters after Chr13 *PRELI* gene targeting (Flox insertion) and Frt-mediated removal of PKG-Neo cassette but before breeding with CD19-Cre mice. Several PCR reaction products from Chr13 *PRELI* ^f/f^ mice are compared to those of Chr13 *PRELI* ^+/f^ and Chr13 *PRELI* ^+/+^ littermates. A *PRELI* gene PCR reaction from CD19Cre mouse DNA is included as an additional Chr13 *PRELI* ^+/+^ control. Predicted sizes of amplified DNA from PRELI^+/+^ and PRELI^f/f^ alleles are indicated. (**c**) Southern blot genotype results of the conditionally targeted mouse strain after breeding with CD19-Cre mice. (Left) Southern blot analysis of BamH1 digested genomic DNA from WT CD19-Cre/Chr13 *PRELI* ^+/+^, heterozygous CD19-Cre/Chr13 *PRELI* ^+/−^ and homozygous CD19-Cre/Chr13 *PRELI* ^−/−^ mice hybridized to the 5′ external probe (illustrated above). Because all mouse lines tested here are in the C57BL/6 genetic background, a WT control from C57BL/6 (BL6) genomic DNA (first lane on the blot) is also included. (Right) Southern blot analysis of EcoR1 and Sal1 digested DNA from heterozygous CD19-Cre/Chr13 *PRELI* ^+/−^, WT CD19/Cre/Chr13 *PRELI* ^+/+^ and homozygous CD19-Cre/Chr13 *PRELI* ^−/−^ mice hybridized to the 3′ external probe (illustrated above). Expected molecular sizes are also indicated.

Gene targeting experiments were undertaken with the aim to inactivate mouse *PRELI* gene expression *in vivo* and define its biological significance. As a means to circumvent potential embryo lethality, a conditional gene-targeting strategy was devised to achieve *PRELI* gene inactivation in a cell lineage-restricted manner. As shown in Figure 3a, flanking loxP (flox)/Cre recognition sites were engineered in the targeting construct in a manner so that sites could be inserted adjacent to exon II of homologous chromosome 13 (Chr 13) *PRELI* gene. In this context, the transfer of exon II with flanking loxP sites (floxed target) into the Chr 13 *PRELI* locus of embryonic stem cells (ESC) could be achieved by homologous recombination. The rationale for loxP sites adjacent to exon II was based on sequence database analyses, which showed that only introns I and II sequences were exempt from regulatory sequences that are transcriptionally relevant to adjacent genes (Fig. 3a). Thus, in agreement with this reasoning, deletion of exon II was designed to interrupt *PRELI* expression without affecting neighboring *Rab24* or *Mad3* (Fig. 3a) gene transcription.

After obtaining mouse lines with homozygous Chr 13 *PRELI* floxed alleles (Chr 13 *PRELI* ^f/f^ mice), as confirmed by polymerase chain reaction (PCR) analyses (Fig. 3b), the animals were bred into the C57BL/6 background to generate and study genetically equal *PRELI* ^f/f^ mouse strains. Although the overall aim in the design and generation of *PRELI* ^f/f^ mice was to provide flexible means to assess the requirement of *PRELI* expression in a broad range of cell lineages, primary breeding with the CD19-Cre transgenic (Tg) mice was chosen for several reasons: (i) *PRELI* was discovered for its prominent expression in GC B cells [1], which are known for their propensity to programmed cell death [32] and hence, it was important to define the impact of its inactivation in these cells; (ii) the successful breeding with CD19-Cre mice is well documented [37], [38]; and (iii) the CD19-Cre mice are readily available from approved animal repositories.

### *PRELI* gene deletion without expression loss

After breeding the Chr 13 *PRELI* ^f/f^ mouse strain with CD19-Cre Tg animals, litters of wild type (WT) CD19-Cre/Chr13 *PRELI* ^+/+^, heterozygous CD19-Cre/Chr13 *PRELI* ^+/−^, and homozygous CD19-Cre/Chr13 *PRELI* ^−/−^ mice were obtained and their genotype routinely verified by Southern blotting (Figure 3c). As a rigorous measure, blots were independently confirmed with external 5′ and 3′ probes, whose respective sequences are located outside the regions of homology with the targeting vector (Fig. 3a).

**Figure 4:**
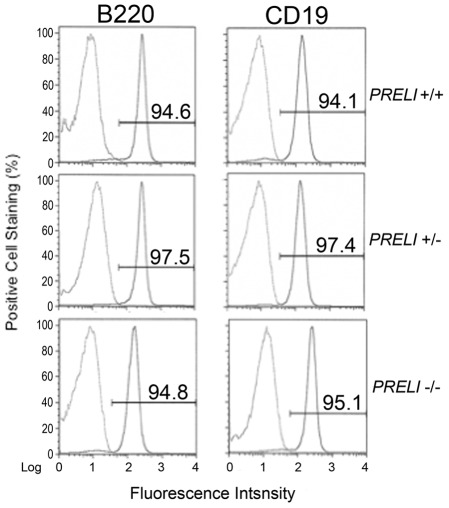
Cell lineage phenotype analyses of CD19Cre/Chr13 *PRELI* mice. Flow cytometry results compare the presence of B220^+^ (left histograms) and CD19^+^ (right histograms) lymphocytes in peripheral blood leukocytes of WT CD19/Cre/Chr13 *PRELI* ^+/+^ (top), heterozygous CD19-Cre/Chr13 *PRELI* ^+/−^ (middle), and homozygous CD19-Cre/Chr13 *PRELI* ^−/−^ mice (bottom). Results are presented as number of cells (%) with relevant fluorescence (Log) staining. The figure is a representative result of at least three independent analyses performed with age-matched littermates (n  =  3). Standard error mean (± SEM) bars are indicated and high statistical significance values scored p≤0.001.

Homozygous CD19-Cre/Chr13 *PRELI* ^−/−^ mice bred normally and did not have any detectable signs of morbidity. *Post mortem* examination of lymphoid organs, from cohorts of euthanized mice, showed no discernible differences in B cell lineage phenotype, homing, or viability between WT CD19-Cre/Chr13 *PRELI* ^+/+^ and homozygous CD19-Cre/Chr13 *PRELI* ^−/−^ littermates. For instance, flow cytometry analyses of CD45/B220^+^ and CD19^+^ cells [34] in peripheral blood leukocytes (PBL) showed that overall leukocyte cellularity (B220) and B lineage-specific (CD19) cell numbers were virtually identical in WT CD19-Cre/Chr13 *PRELI* ^+/+^, heterozygous CD19-Cre/Chr13 *PRELI* ^+/−^, and homozygous CD19-Cre/Chr13 *PRELI* ^−/−^ mice (Fig. 4). Furthermore, analyses of offspring resulting from the breeding of *PRELI* ^f/f^ mouse lines with ubiquitous CMV-driven Cre expressing mice gave similar results as those obtained with CD19-Cre (not shown).

**Figure 5:**
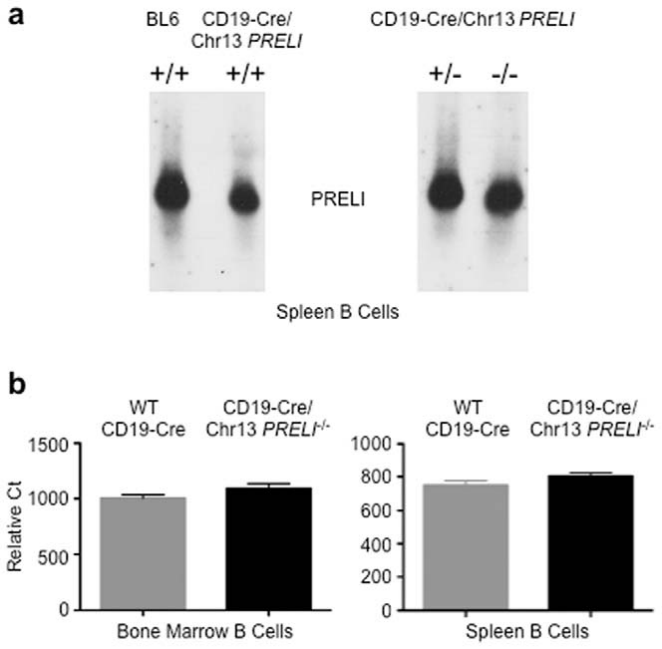
*PRELI* transcription by CD19Cre/Ch13 *PRELI* mice. (**a**) Northern blots compare PRELI mRNA levels between WT CD19/Cre/Chr13 *PRELI* ^+/+^ and heterozygous CD19-Cre/Chr13 *PRELI* ^+/−^ or homozygous CD19-Cre/Chr13 *PRELI* ^−/−^ mouse spleen B cells. (**b**) qRT-PCR assessment of *PRELI* transcription between WT CD19/Cre/Chr13 *PRELI* ^+/+^ and homozygousCD19-Cre/Chr13 *PRELI* ^−/−^ mice. qRT-PCR comparisons include results obtained with RNA from bone marrow and spleen B cells. Data are presented in relative Ct and are the result of at least three WT CD19/Cre/Chr13 *PRELI* ^+/+^ and CD19-Cre/Chr13 *PRELI* ^−/−^ paired littermates.

**Figure 6:**
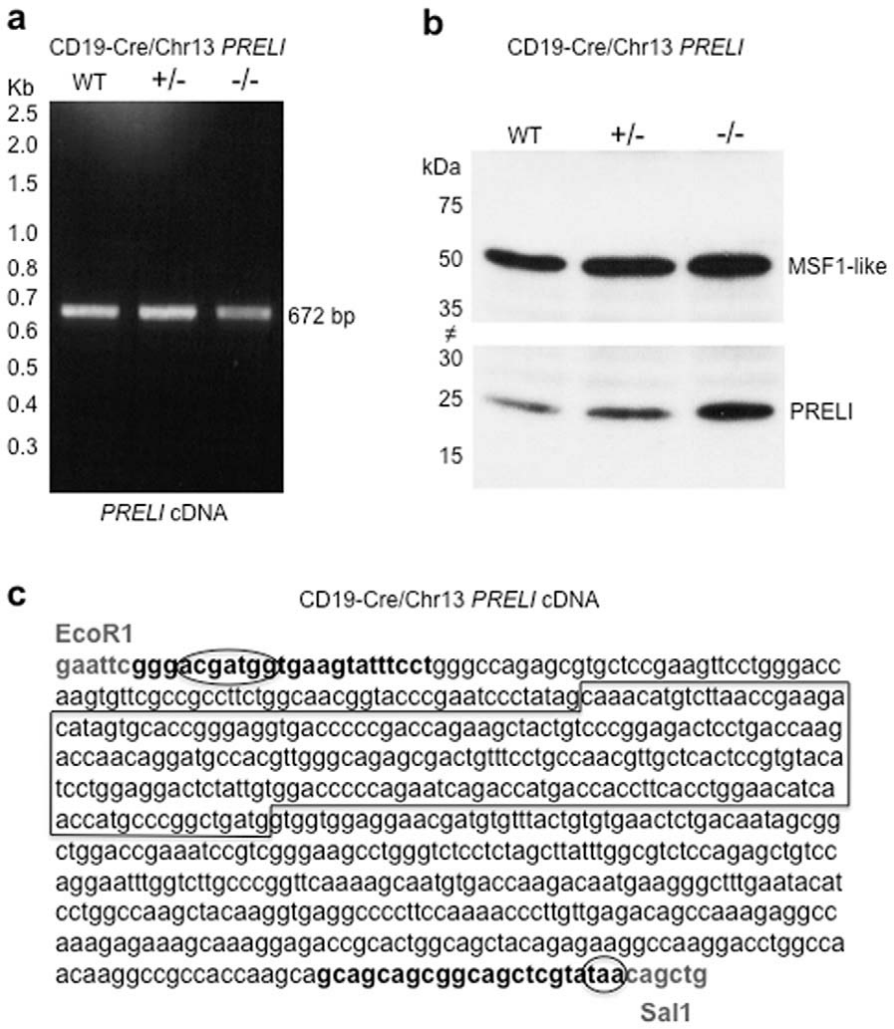
CD19-Cre/Ch13 *PRELI* ^−/−^ transcripts have exon II sequences. (**a**) RT-PCR results of peripheral blood leukocyte (PBL) *PRELI* transcript amplification from WT CD19/Cre/Chr13 *PRELI* ^+/+^, heterozygous CD19-Cre/Chr13 *PRELI* ^+/−^, and homozygous CD19-Cre/Chr13 *PRELI* ^−/−^ mouse littermates. A 2.5–0.3 kb molecular weight marker range is shown on the left of the ethidium bromide-stained gel image to verify the length of *PRELI* open reading frame (ORF). The predicted 670 bp molecular size of *PRELI* ORF PCR fragments includes forward and reverse amplifying primers with respective restriction endonuclease EcoR1 and Sal1 sequences (depicted in section c). (**b**) Immunoblot results reveal PBL PRELI protein levels in WT CD19/Cre/Chr13 *PRELI* ^+/+^, heterozygous CD19-Cre/Chr13 *PRELI* ^+/−^, and homozygous CD19-Cre/Chr13 *PRELI* ^−/−^ mice. Of note, the same blot was divided into high and low molecular mass (kDa) sections, indicated by the intersect mark (≠) at proportional kDa boundaries. This strategy enables the assessment of PRELI expression (24.9 kDa) and at the same time, permits to independently monitor protein loading (MSF1-like) on identical sample lanes. (**c**) cDNA sequencing results obtained from a representative homozygous CD19-Cre/Chr13 PRELI^−/−^ clone obtained after RT-PCR amplification (section a). Amplifying forward and reverse primers are shown in bold font and respective EcoR1 and Sal1 restriction endonuclease target sequences are indicated in grey font. Canonical translation initiation and stop codon sequences are circled, while exon II is framed. This sequence is identical to NCBI database annotation XM_001476721.

Although no other LEA genes with similar *PRELI* structure and mechanisms of action have been identified in mammals, functional redundancy with other gene families was plausible. Therefore, defining mRNA and protein expression status in CD19-Cre/Chr13 *PRELI* KO mice was imperative, before functional overlap could be concluded. Strikingly, northern blot and quantitative reverse transcription-mediated PCR (qRT-PCR) [39], [40], [41] revealed that *PRELI* mRNA expression in bone marrow and spleen B cells was virtually unaffected in homozygous CD19-Cre/Chr13 *PRELI* ^−/−^ mice (Fig. 5a and b), despite proven Chr13 *PRELI* gene deletion (Fig. 3b and c). Moreover, northern blot results showed no discernible differences in *PRELI* mRNA molecular sizes between WT CD19-Cre/Chr13 *PRELI* ^+/+^ and homozygous CD19-Cre/Chr13 *PRELI* ^−/−^ littermates, even though exon II spans nearly one fourth (226 bp) of the entire 1.1 kb transcript sequence. Nevertheless and although Chr13 *PRELI* gene targeting was engineered to completely prevent transcription, exon II-skipping, which could generate truncated *PRELI* transcripts and proteins required to be ruled out. To that end, amplification of full-length PRELI open reading frame (ORF), directional cDNA cloning, and immunoblotting experiments were performed using RNA and protein extracts from CD19-Cre/Chr13 *PRELI* KO peripheral blood leukocytes (PBL), to verify *PRELI* mRNA and protein molecular sizes and examine the nucleotide sequence of the amplified ORF. Figure 6 demonstrates that the amplified 672 ORF (Fig. 6a), the 25-kDa polypeptide (Fig. 6b), and the exon II-retaining full-length cDNA sequence (Fig. 6c) derived from RNA and protein extracts of CD19-Cre/Chr13 *PRELI* KO PBL, encode intact *PRELI* gene products.

These results, together with the evidence of conditional Cre-mediated Chr13 *PRELI* gene deletion (Fig. 3b and c) suggested that the PRELI mRNA and protein expression observed in homozygous CD19-Cre/Chr13 *PRELI* ^−/−^ mice could not have originated from the Chr 13 locus. It is therefore conceivable that complementary loci, encoding *LEA* proteins of related or identical gene pool, could operate in concert to overcome Chr13 *PRELI* deficiency.

### Spare *PRELI* loci compensate the Chr 13 *PRELI* gene deficiency

**Figure 7:**
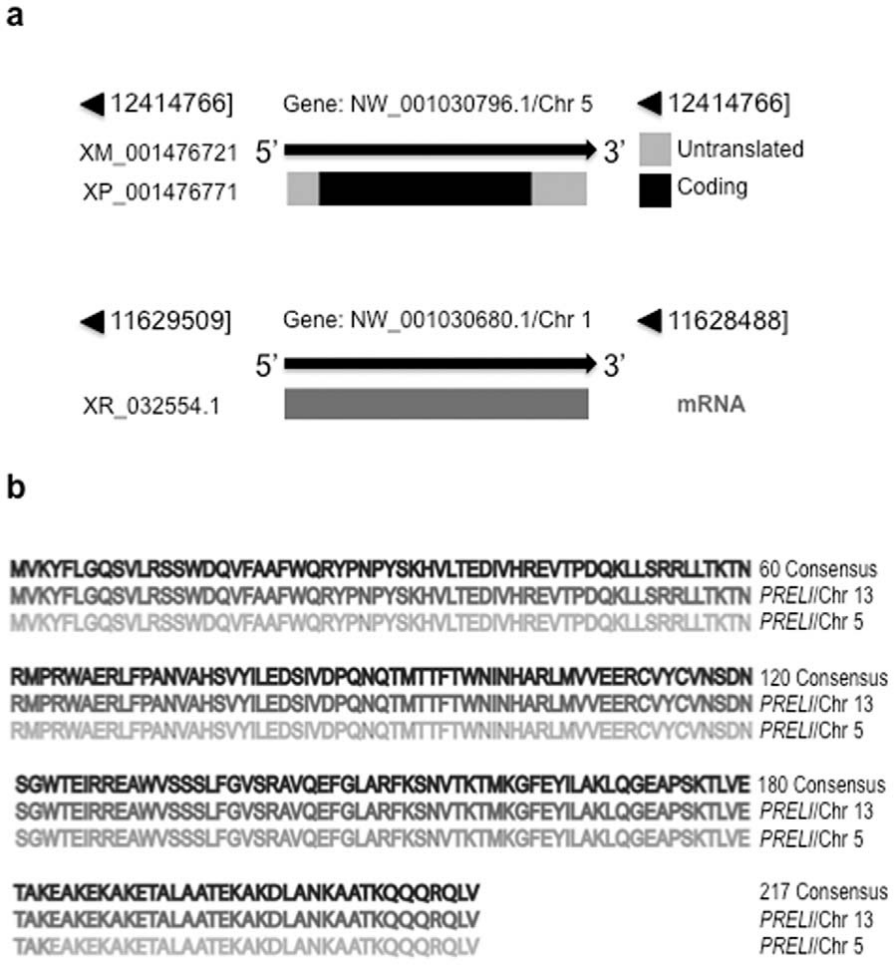
Additional *PRELI* gene copies in Chr 5 and 1 loci. (**a**) Top: Relative *PRELI* gene location in Chr 5 is shown with distance/direction coordinates (black triangles) and accession number; transcript database annotation at (http://www. ncbi.nlm.nih.gov/sites/) is provided; 5′-3′ transcript orientation with respect to the gene locus is indicated by a thick black arrow; and protein annotation, which also originated from the same uniform resource locator (URL) link shown above, depicts deduced 5′ and 3′ untranslated regions (in grey) and coding open reading frame (in black). Bottom: Designated *PRELI* gene location in Chr 1, which includes distance/direction coordinates (black triangles) and database accession number. It also displays database annotation of respective transcript at (http://www.ncbi.nlm.nih.gov/sites) and its relative 5′-3′ direction with respect to the gene locus is shown by a thick black arrow. A region spanning the predicted mRNA is shown as a grey rectangle. (**b**) Amino acid sequence alignment between proteins respectively encoded by Chr 13 and Chr 5 *PRELI* gene loci is shown in color-coded format: A numbered consensus generated by the Clustal alignment software (see methods) is shown in black font, while Chr 13 PRELI and Chr 5 PRELI amino acid sequences are respectively aligned in dark and grey font.

In support of this hypothesis, updates of mouse databases revealed the presence of additional *PRELI* gene copies in mouse chromosome 5 and 1. Strikingly, *PRELI* copy in Chr5 is an intronless gene (Fig. 7a) and transcribes mRNAs, whose deduced amino acid sequence is 100% identical to that encoded by the Chr13 *PRELI* gene (Fig. 7b). Similarly, Chr1 *PRELI* gene is also intronless (Fig. 7a) but its nucleotide sequence is only 98% identical to Chr5 and 13 genes (not shown). The distinctive nucleotide divergence of the Chr1 *PRELI* gene from those of Chr5 and 13 loci appears to give rise to premature stop codons, which would preclude *PRELI*-like translation. However, the annotated nucleotide sequence data was generated from pooled DNA of diverse mouse strains and hence, the likelihood of strain-related polymorphisms and/or nucleotide mismatches cannot be completely excluded. Nevertheless, the prominent *PRELI* mRNA and protein levels found in all homozygous CD19-Cre/Chr13 *PRELI* ^−/−^ mouse lines examined indicate that overall expression is amply sufficient to surmount Chr13 *PRELI* gene deficiency, irrespective of whether it originated from Chr5 *PRELI* alone or from combined Chr5 and Chr1 *PRELI* gene transcription. Moreover, the compensatory *PRELI* mRNA and protein expression in CD-19Cre/Chr 13 PRELI^−/−^ mice was vastly kept at physiological levels. Therefore, retention of B cell responses to stress and death signaling is anticipated. These findings additionally suggest that inter-chromosomal *PRELI* gene duplication represent evolutionarily conserved *LEA*-dependent mechanisms to preserve vital physiology.

In further support of the relevance of *in vivo PRELI* functions, recent studies have demonstrated that transgenic (Tg) mice ubiquitously expressing high levels of mutant PRELI proteins that lacked the functional *LEA* motif (*PRELI*/LEA^−^) succumbed at early embryo stages [5]. *PRELI*/LEA^−^ Tg embryos died of massive apoptosis, caused by a dominant-negative molecular hindrance, which prevented endogenous *LEA*-dependent cytoprotection and severely compromised mouse development [5]. Moreover, Tg mice in which mutant *PRELI*/LEA^−^ expression was driven by the endothelial and hematopoietic cell lineage-specific vav promoter [42], [43], died of systemic haemorrhages within five weeks of postnatal life and showed poor lymphoid organ development [5]. Remarkably, no other proteins with similar tissue distribution, mitochondria localization and pro-survival functions could circumvent *PRELI*/*LEA* ^−^ Tg lethality, thus emphasizing the unique *LEA-*dependent mechanisms that are essential for plant and animal embryo development and cell survival [3], [4], [5], [18], [20], [21], [23], [30], [31].

Thus, the present work emphasises the conserved cytoprotection activity of LEA-containing proteins in general [1], [3], [4], [5], [18], [20], [21], [28] and supports the vital PRELI physiology, which could be upheld by spare chromosome imprints that confer failsafe genetic means to ensure the survival of cells undergoing strenuous development [20], [21], [28], [30], [31] and selection pressure [5], [32], [44].

## Discussion

Eukaryote cells react to stress and death-inducing stimuli by a coordinated transduction of signals that trigger the expression of genes that are relevant for cell homeostasis and survival [20], [21], [30], [45]. In keeping with this notion, *LEA* motif-containing genes encode an evolutionarily conserved family of proteins, which have emerged as archetypal protectors of eukaryote cells against stress and death cues [3], [4], [18], [20], [21], [23], [28], [30], [31]. Although *LEA* proteins were first identified and studied in plants [19], [28], [30], [45], [46], a decade of broad prokaryote and eukaryote research emphasizes their evolutionary conservation and biological impact in stress tolerance [1], [5], [20], [21], [31], [47]. For instance, the *Drosophila* proteins *prel* (*PRELI-like*), *slmo* (*slowmo*) and *retm* (*real-time*) proteins constitute a family of mitochondrial *LEA*-containing orthologs, which also possess conserved MSF1-like domains and are activated during embryo development [3], [23]. Analogous to plants, nematodes use *LEA*-dependent anhydrobiosis functions as protective means against dehydration [20], [48]. Moreover, *Ups1p* is a yeast *LEA* protein that is localized within mitochondrial intermembrane space (IMS), whose known interactions with *Mgm1p* keep mitochondria cristae junctions tight and prevent the exit of apoptogenic molecules [4], [17].

As the first example of *LEA*-containing proteins in vertebrates, the *PX19* gene is an avian ortholog, whose expression is induced in response to stress-inducing bromodeoxyuridine and appears to be critical for haematopoietic cell development [18]. Among vertebrates, *PRELI* is a mammalian *LEA*-containing protein with N-terminal mitochondrial localization MSF1-like signals and tandem C-terminal repeats of the *LEA* domain [1], [2]. Identical to yeast *Ups1p*, *PRELI* locates within the IMS and interacts with *OPA1*, the mammalian homolog of yeast *Mgm1p* [6], [22], to uphold mitochondria membrane potential Δψ_m_, support oxidative phosphorylation reactions and regulate protein traffic [5]. The evidence that human *PRELI* can biochemically and functionally substitute yeast *Ups1p* in the mitochondria [17], further emphasizes their biological resemblance.

In view of the widespread support of *LEA* protein physiology in eukaryote cell protection against death-inducing stimuli [1], [3], [4], [18], [20], [21], [28], [30], [31] and given the robust *PRELI* transcript levels during embryo development [5], the rational inference was that *LEA* gene expression could be vital in mammals. Consistent with this hypothesis, Tg mice with dominant-negative *PRELI/LEA* ^−^ expression died at early embryo stages or soon after birth, as a result of molecular hindrance preventing endogenous *PRELI* functions [5]. Furthermore, additional *PRELI* gene copies in mouse Chr 5 and 1 have been identified (Fig. 7), whose expression seems sufficient to keep mRNA and protein expression at physiological levels and thus overcome Chr13 *PRELI* gene loss.

Relevant to the safeguard of essential biology, *LEA* proteins in plants and animals are recognized for their multiple gene copy distribution within the same organism [31]. In plants, fourteen of the 51 *LEA* genes, grouped into nine classified families, are duplicated [49], [50]. The imprinted *LEA* genetics for physiological safety can therefore be interpreted as evolutionarily conserved means to keep organelle structures intact and thus ensure cell functions and survival. These include, maintenance of mitochondria cristae junctions to support RC oxidative phosphorylation reactions, uphold osmolarity, regulate ROS levels, prevent dehydration, and protect cells against thermodynamic shock-induced stress [5].

Remarkably, *LEA* gene duplication is not restricted to plants [31] and thus, the relatively homogenous distribution of spare copies either within the same or distinct chromosomes [50] likely reflects conserved measures to bank essential genetics, via inter and intra-chromosome gene repeats. In nematodes, homologous av*LEA* and ce*LEA* proteins regulate tolerance to desiccation-induced stress and overall protein stabilization functions [20], [21]. Although identical *LEA* gene duplicates are yet to be identified in *Drosophila*, this species counts on the *PRELI*-like proteins *Slmo*, *Prel* and *Retm* to home in the mitochondria and sustain RC-dependent ATP synthesis [3], [23]. In the same context, yeast *Ups1p* and *Ups2p* share remarkable resemblance with *PRELI* in supporting mitochondria structure integrity and physiology [4], [17].

The present study speculates that *LEA* proteins in mammals possess gene duplication traits equivalent to those observed in plants [31], [49], [50]. Consistent with this theory, gene and protein databases have revealed that the mouse carries at least two spare *PRELI* gene copies in Chr 5 and Chr 1 (Fig. 7). Importantly, the probability of persistent *PRELI* expression, originating from duplicate gene loci is high for Chr 5 *PRELI* and conceivable for Chr 1 *PRELI* genes. It is therefore plausible that gene expression from Chr 5 *PRELI* or in conjunction with Chr 1 *PRELI* locus occurs as a means to preserve functions that are essential for mouse embryo development and cell survival. However, it must be noted that although the presence of spare *PRELI* genes represent backup means to circumvent the loss of the Ch 13 locus, expression in CD19-Cre/Ch 13 *PRELI* ^−/−^ mouse B cells remains under the same genetic and epigenetic regulatory mechanisms as those of WT B cells. In this context B cell *PRELI* expression is also under physiologic control of cell development and maturation signals.

**Figure 8:**
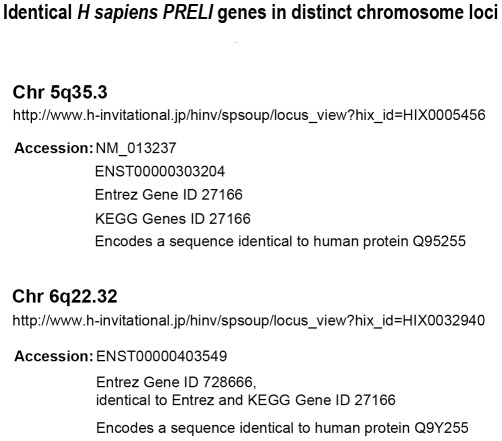
Spare *PRELI* gene copies in humans. This figure provides mRNA and protein database URL information, including gene ID annotations, chromosome coordinates, nucleotide and protein accession number annotations for human PRELI gene duplicates found in Chr 5q35.3 and Chr 6q22.32.

In conclusion, the data presented and discussed in this study highlight the biological significance of LEA proteins in general and PRELI in particular, whose physiology links mitochondria biogenesis, energy metabolism, and cell responses to stress and death signaling [5]. The impact of these functions, which involve hallmark mechanisms relevant to embryo organogenesis, development and survival in plants and animals [3], [4], [5], [17], [18], [20], [21], [28], [30], stems from the remarkable array of LEA proteins across evolution and the inherent capacity to undergo gene locus duplication [31], [49], [50]. In keeping with this concept, LEA genes epitomize conserved molecular models that have been long presumed to portray backup genetics applied to mechanisms of cell preservation [49], [50]. The fact that mouse *PRELI* gene undergoes inter-chromosomal gene duplication provides experimental support to such prediction and suggests that spare Chr 5 and potentially Chr 1 *PRELI* loci (Fig. 7) play genetically meaningful roles to uphold cytoprotection functions after Chr 13 *PRELI* gene loss. Lastly and to underscore the relevance of the evolutionary conservation of *LEA* duplication traits, figure 8 summarizes gene database annotations, as evidence that human *PRELI* gene also undergoes inter-chromosomal duplication at Chr 5q35.3 and Chr 6q22.32. Notably, human Chr 5 and 6 *PRELI* loci are confirmed spare gene copies, which encode amino acid sequence that are 100% identical to the Q9Y225 protein annotation (Fig. 8).

Although future research will provide full appraisal of *PRELI* biological significance, the present study aims to share information deemed relevant to the broad Biology field.

## Materials and Methods

### Cloning and Sequencing

A mouse spleen cDNA library (Clontech Laboratories, Inc., Palo Alto, CA) was screened by hybridization to a human *PRELI* cDNA probe. A selected clone was purified, sequenced (DNA Sequencing Core Facility, UT-MDACC) and found to be identical to an annotated sequence in the National Center for Biotechnology Information (NCBI) database: Accession # XM_001476721. The sequence information served to retrieve a Chr 13 bacterial artificial chromosome (BAC) clone (Accession # NT_039589), which was used as the base to design the strategy for conditional *PRELI* gene targeting in mice.

### *PRELI* mRNA Expression

*PRELI* mRNA *in situ* hybridization was performed on normal 13.5 dpc embryo sections, covered with 4 million counts of ^35^S-UTP-labeled antisense or sense complementary RNA (cRNA) probes and hybridized overnight (ON) at 60°C. Post-hybridization washes were processed as previously reported [51]. After ON incubation, slides were dipped in Kodak NTB-2 emulsion and exposed for three days. Images were acquired using an Olympus BX60 microscope equipped with a SPOT digital camera (Diagnostics Instruments). Also, embryo stage-specific (days post coitum or dpc.) and multiple tissue northern (MTN) blots (Clontech, Palo Alto CA), routinely normalized against housekeeping transcripts, were respectively hybridized to ^32^P-labeled mouse *PRELI* cDNA probes, as recommended by the manufacturer.

### Confocal Fluorescence Microscopy

#### For sub-cellular *PRELI* protein localization

Control and *PRELI* Blin-1 transfectants [5] were centrifuged (800 rpm) onto slides and immediately fixed with 4% paraformaldehyde. Slides were probed with anti-HSP60 monoclonal antibody (Stressgen Biotechnologies, Victoria, BC, Canada) in combination with custom-produced anti-*PRELI* polyclonal IgGs (Genemed Synthesis, Inc., San Antonio, TX). Anti-*Hsp60* and *PRELI* antibody reactivities were respectively revealed by green fluorescence (Alexa 488)-conjugated anti-mouse and red fluorescence (Alexa 594)-conjugated anti-rabbit IgGs (Molecular Probes, Eugene, OR).

#### For mitochondria morphology and *AIF* release analyses

Control and *PRELI*/Blin-1 transfectants [5] were treated with or without 1 µM STS. After an 8 hr treatment, cells were harvested, placed onto polylysine-coated slides (CEL Associates, Pearland, TX) and fixed with 4% paraformaldehyde. The fixed cells were probed with polyclonal anti-AIF (Chemicon International, Temacula, CA) IgGs. Polyclonal anti-AIF reactivity was revealed by red fluorescence (Alexa 594)-conjugated anti-rabbit IgGs (Molecular Probes, Eugene, OR). Images were captured by confocal microscopy (Olympus 1X71; PMT 0-900v; Scan speed 0.005184 sec/line). To emphasize organelle/cell morphology and protein distribution red fluorescence, AIF images were mounted against greyscale cell background using Adobe Photoshop CS4 (Adobe Systems Inc. San Jose, CA) software.

### Generation of CD19-Cre/Chr 13 *PRELI* deficient Mice

To generate conditional *PRELI* deficient mouse lines, the targeting vector EASY-FLIRT was used to insert Cre recombinase loxP recognition sequences into the Chr 13 *PRELI* gene locus (Fig. 3a) in a manner so that exon II was flanked by two LoxP sites (Floxed target). The mouse *PRELI* gene on chromosome 13 was replaced by homologous recombination in 129 embryonic stem cells. After obtaining homozygous *PRELI* ^f/f^ mouse lines, animals were extensively backcrossed into the C57BL/6 background to produce genetically equal *PRELI* ^f/f^ mouse strains. This approach aimed to facilitate subsequent breeding schemes with most C57BL/6-Cre mice. Wild type and heterozygous littermates were routinely used as controls. Genotyping of the engineered mouse lines was confirmed by PCR and Southern blot analyses. The forward 5′cttcaccctgatgctgcacgggt c3′and reverse 5′accgaggcacaccccagttatct3′ primer sequences that were used in the design of PCR based experiments to assess the genotype of floxed *PRELI* mouse litters are respectively located at relative *PRELI* genomic positions 4298–4323 (within exon I) and 5761–5784 (intron II). The 5′ (*Rab24* gene locus) and 3′ (*Mad3* locus) external probes, as indicated in Figure 3a, were used in confirmatory Southern blot analyses.

### Quantitative Reverse Transcription (RT)-mediated PCR (qRT-PCR)

Pure RNA preparations from bone marrow, peripheral blood leukocytes (PBL) and spleen cells were obtained to quantitatively compare *PRELI* mRNA expression levels between WT CD19-Cre/Chr13 *PRELI* ^+/+^ and homozygous CD19-Cre/Chr13 *PRELI^−/−^* B cells using SABioscience/QIAGEN (Frederick, MD) reagents and RT2qRT-PCR primer assay kit (Catalogue # PPM40154). qRT-PCR reactions were carried out as reported elsewhere [39], [40], [41], using SYBR Green/Rox PCR master mix (Sabioscience, Frederick, MD, USA) in a 7500 fast real time PCR system (Applied BioSystems, Carlsbad, CA).

### *PRELI* transcript amplification, cloning and sequencing

Total RNA was purified from PBL of WT CD19-Cre/Chr13 *PRELI* ^+/+^, heterozygous CD19-Cre//Ch13 *PRELI* ^+/−^, and homozygous CD19-Cre/Ch13 *PRELI* ^−/−^ mouse littermates by standard procedures. RNA was reverse-transcribed using the Superscript II RT kit (Invitrogen, Carlsbad, CA) according to the manufacture's protocol. The *PRELI* open reading frame was subsequently amplified by the high fidelity DNA polymerase method (Roche, Mannheim, Germany) using forward 5′aagctt**gaattc**gggacgatggtgaagtatttcct3′ and reverse 5′ggtacc**gtcgac**tatacgagctgccgctgctgctg3′ primers that respectively included EcoR1 and Sal1 (bold font) to facilitate directional cloning. RT-PCR products were gel-purified, cloned into the pCMV-Script vector (Stratagene-Agilent Technologies/Genomics, Santa Clara, CA), automatically sequenced (The University of Texas MD Anderson Sequencing Core), and aligned for Lasergene (DNAstar, Inc. Madison, WI) sequence analysis.

### Immunoblotting

10 µg of PBL protein lysates, obtained from each WT CD19-Cre/Chr 13 *PRELI* ^+/+^, heterozygous CD19-Cre/Chr 13 *PRELI* ^+/−^, and homozygous CD19-Cre/Chr13 *PRELI* ^−/−^ mice were each resolved by 12% SDS-PAGE and electro-transferred overnight onto a nitrocellulose matrix. The blot was divided into high (above the 35 kDa mark) and low (below the 35 kDa mark) molecular sections and respectively reacted overnight, at 4°C, to custom-produced polyclonal anti-MSF1-like and *LEA* sequence-specific anti-*PRELI* IgGs (Bethyl Laboratories, Montgomery, TX). After intense washing, reactions were revealed with HRP-conjugated anti-rabbit IgG (Abcam) and chemilluminiscence (Pierce, Rockford, IL) autoradiography, according to the manufacturer instructions.

### Ethics Statement

All mice in this study were housed under specific pathogen-free conditions and experiments were conducted according to approved Institutional Animal Care and Use Committee (IACUC) protocol # 03-06-03632.
